# Pulmonary ORMDL3 is critical for induction of *Alternaria*-induced allergic airways disease

**DOI:** 10.1016/j.jaci.2016.07.033

**Published:** 2017-05

**Authors:** Stephan Löser, Lisa G. Gregory, Youming Zhang, Katrein Schaefer, Simone A. Walker, James Buckley, Laura Denney, Charlotte H. Dean, William O.C. Cookson, Miriam F. Moffatt, Clare M. Lloyd

**Affiliations:** a Inflammation, Repair & Development Section, National Heart and Lung Institute, Imperial College London, London, United Kingdom; b Genomic Medicine Centre, National Heart and Lung Institute, Imperial College London, London, United Kingdom

**Keywords:** ORMDL3, asthma, unfolded protein response, uric acid, *Alternaria*, AAD, Allergic airways disease, AAV, Adeno-associated viral vector, AHR, Airway hyperreactivity, ATF, Activating transcription factor, BALF, Bronchoalveolar lavage fluid, EGFP, Enhanced green fluorescent protein, ER, Endoplasmic reticulum, GWAS, Genome-wide association study, IRE, Inositol requiring ER-to-nucleus signal kinase, KO, Knockout, ORMDL, ORM-1 like protein, PERK, Double-stranded RNA-activated protein kinase (PKR)-like endoplasmic reticulum kinase, SERCA2b, Sarco-endoplasmic reticulum Ca^2+^ ATPase 2b, SNP, Single nucleotide polymorphism, UK, United Kingdom, UPR, Unfolded protein response, WT, Wild-type, *Xbp1*, X-box binding protein 1

## Abstract

**Background:**

Genome-wide association studies have identified the ORM (yeast)-like protein isoform 3 (*ORMDL3*) gene locus on human chromosome 17q to be a highly significant risk factor for childhood-onset asthma.

**Objective:**

We sought to investigate *in vivo* the functional role of ORMDL3 in disease inception.

**Methods:**

An *Ormdl3*-deficient mouse was generated and the role of ORMDL3 in the generation of allergic airways disease to the fungal aeroallergen *Alternaria alternata* was determined. An adeno-associated viral vector was also used to reconstitute ORMDL3 expression in airway epithelial cells of *Ormdl3* knockout mice.

**Results:**

*Ormdl3* knockout mice were found to be protected from developing allergic airways disease and showed a marked decrease in pathophysiology, including lung function and airway eosinophilia induced by *Alternaria. Alternaria* is a potent inducer of cellular stress and the unfolded protein response, and ORMDL3 was found to play a critical role in driving the activating transcription factor 6–mediated arm of this response through *Xbp1* and downstream activation of the endoplasmic reticulum–associated degradation pathway. In addition, ORMDL3 mediated uric acid release, another marker of cellular stress. In the knockout mice, reconstitution of *Ormdl3* transcript levels specifically in the bronchial epithelium resulted in reinstatement of susceptibility to fungal allergen–induced allergic airways disease.

**Conclusions:**

This study demonstrates that *ORMDL3*, an asthma susceptibility gene identified by genome-wide association studies, contributes to key pathways that promote changes in airway physiology during allergic immune responses.

## Graphical abstract

**Figure:**
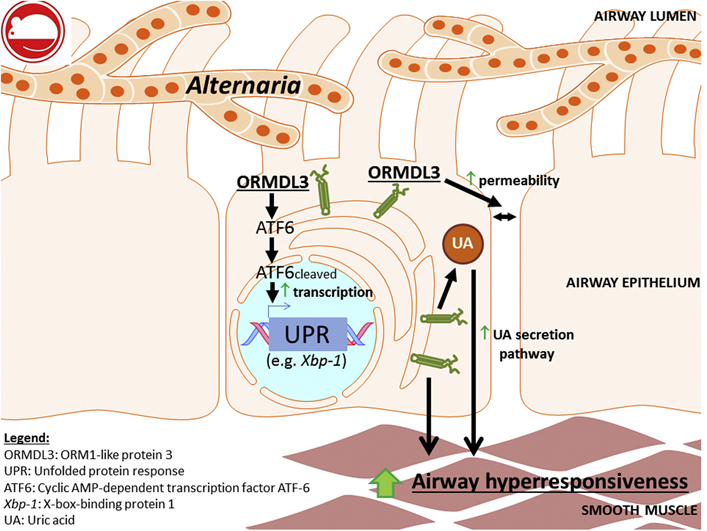

Asthma encompasses a range of pulmonary disease phenotypes commonly defined by a type 2 inflammatory response to airborne allergen concomitant with airway hyperresponsiveness (AHR).^1^ Although the underlying etiologies for asthma are incompletely understood, it is recognized that the epithelial barrier plays a central role in asthma pathogenesis and that genetic predisposition can be pivotal for asthma development.^2^ A pioneering genome-wide association study (GWAS) identified single nucleotide polymorphisms (SNPs) on chromosome 17q21 that were strongly linked to asthma.^3^ The same SNPs were also found to be associated with increased expression of transcripts of the *ORM-1* like protein 3 (*ORMDL3*) gene.^3^ However, the functional role of ORMDL3 in asthma has not as yet been fully elucidated, and it is not known how this molecule contributes to disease pathophysiology.

ORMDL3 is expressed in various tissues, including the lung where it is predominantly localized to airway epithelial cells in response to *Alternaria* or ovalbumin challenge.^4^, ^5^ It is a transmembrane protein located in the endoplasmic reticulum (ER)^4^ where physiologically it acts to negatively regulate sphingolipid synthesis.^6^ Intriguingly, it has been demonstrated that reduction of sphingolipid synthesis with pharmacological compounds increases AHR in mice and human bronchial tissue.^7^ However, in a murine model of house dust mite–induced allergic airways disease (AAD), elevated levels of ORMDL3 in response to allergen correlate with increased ceramide production.^8^ Previous studies with mice universally overexpressing ORMDL3 have demonstrated that these mice spontaneously develop increased AHR and airway remodeling, which precedes elevated type 2 pulmonary inflammation.^9^

ER stress can be induced on perturbation of luminal Ca^2+^ levels within the ER, consequently decreasing the efficiency of correct protein folding.^10^ Accumulation of nascent or misfolded protein in the ER lumen leads to activation of the unfolded protein response (UPR).^11^, ^12^ Available data indicate that ORMDL3 modulates the UPR although the pathways involved may be tissue specific. Bone marrow–derived macrophages from *Ormdl3* transgenic mice have increased activation of the activating transcription factor (ATF) 6 pathway of the UPR,^9^ similar to previous observations using lung epithelial A549 cells transfected with *ORMDL3 in vitro*.^5^ In these systems, expression of ORMDL3 is positively correlated with expression of sarco-endoplasmic reticulum Ca^2+^ ATPase 2b (SERCA2b), a downstream target of the ATF6 pathway.^13^ In HEK293 and Jurkat cells transfected with *Ormdl3*, the data illustrate that ORMDL3 binds to and inhibits SERCA, thereby impeding entry of cytosolic Ca^2+^ into the ER lumen and initiating the UPR.^14^ However, within this experimental setting, the main impact of ORMDL3 is on the double-stranded RNA-activated protein kinase (PKR)-like endoplasmic reticulum kinase (PERK)/eIF2α arm of the UPR.

In the present study, *Ormdl3*-deficient mice were generated to investigate the role of ORMDL3 in determining the pulmonary response to allergen. In addition, we have reconstituted ORMDL3 specifically in the bronchial epithelium of these knockout (KO) mice to ascertain the specific contribution of epithelial ORMDL3. These mice reveal that ORMDL3 is pivotal in the generation of fungal allergen–induced AHR via modulation of cellular stress pathways. Our data reinforce the findings of asthma GWAS, placing ORMDL3 as an important mediator in the development of allergen-induced AHR.

## Methods

### Generation of *Ormdl3* KO mice

**Fig E1:**
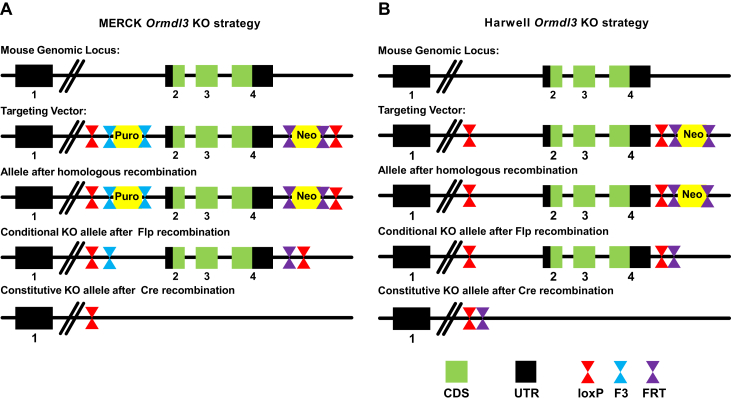
Comparative schematic of strategies used to generate *Ormdl3*^MER^ KO and *Ormdl3*^HAR^ KO mice. **A,** Strategy used by MERCK. The gene *Ormdl3* contains 4 exons of which exons 2 to 4 are encoding the ORMDL3 protein. Exons 2 to 4 were consequently flanked by 2 antibiotic selection cassettes (Puro, puromycin; Neo, neomycin) and loxP recombination sites were inserted upstream of the Puro cassette and downstream of the Neo cassette. Following homologous recombination, Flp recombination, and Cre recombination steps, the resulting *Ormdl3*^MER^ KO strain retains a loxP recombination site downstream of exon 1. **B,** The approach used by MRC Harwell slightly differed from the MERCK strategy, as a single neomycin selection cassette was included in the targeting vector. LoxP recombination sites were located between exons 1 and 2 and between exon 4 and the FRT-flanked Neo cassette. The resulting *Ormdl3*^HAR^ KO mice strain therefore retains a loxP, as well as an FRT recombination site after homologous, Flp and Cre recombination. *CDS*, Coding sequence; *UTR*, untranslated region.

Two separate *Ormdl3* alleles were used in the study. The first was generated by Dr. Y Zhang at the Mary Lyon Centre, MRC Harwell, and the second was made and kindly donated by Merck Research Laboratories. Both constructs contained LoxP sites flanking exons 1 to 4 of *Ormdl3*, the translation initiation site of the gene being contained within exon 2. Initially, conditional alleles were generated by Flp-mediated removal of selection cassettes. The targeting strategies for both lines were similar; however, the Merck construct contained dual Neo and Puro selection cassettes, whereas the Harwell allele contained a single Neo selection cassette (see Fig E1 in this article's Online Repository at www.jacionline.org). At Merck, targeting constructs were micro-injected into embryonic stem cells from C57/Bl6NTac mice and complete *Ormdl3* KOs were subsequently generated by Cre-mediated recombination, using globally expressed Cre-driver mouse lines.^15^ At Harwell, targeting constructs were micro-injected into ES cells from R1 129 ES cells and *Ormdl3* KOs were subsequently generated by Cre-mediated recombination, using Tg(ACTB-cre)3Mrt mice (Infrafrontier; accession no. EM:06107). These *Ormdl3* KO mice were subsequently crossed on to a C57BL/6J background (10 generations). A similar response to allergen was observed in both strains. The MERCK *Ormdl3* KO line (*Ormdl3* KO^Mer^) was used for experiments where mice were treated as adults. Experiments which required in-house breeding at Imperial College were performed using the *Ormdl3* KO line generated by Dr Y Zhang (*Ormdl3* KO^Har^).

### Construction of enhanced green fluorescent protein and *Ormdl3*-enhanced green fluorescent protein adeno-associated viral vectors

An *Ormdl3-*enhanced green fluorescent protein (*EGFP*) (N-terminal tag) gene fusion was constructed and cloned into plasmid pZac2.1 under control of the ubiquitous cytomegalovirus promoter. Adeno-associated viral vectors (AAVs) were produced by Penn Vector Core (University of Pennsylvania, Pa). On day 5 of life, neonatal mice were intranasally administered 1 × 10^11^ genome copies of either AAV *EGFP* (control vector) or AAV *Ormdl3-EGFP* (AAV *Ormdl3*) with 50 mU neuraminidase (Sigma-Aldrich, Dorset, United Kingdom [UK]) in PBS. At age 8 weeks, mice received either 10 μg *Alternaria alternata* extract (Greer Laboratories, Lenoir, NC) or PBS via intranasal instillation, 3 times per week for 5 weeks. Mice were killed 18 hours postfinal *Alternaria* instillation.

### Induction of AAD

Ten- to 12-week-old male wild-type (WT) and *Ormdl3* KO^Mer^ mice were bred at Taconic Biosciences (Germantown, New York, NY). WT and *Ormdl3* KO^Har^ mice (Harwell, UK) were bred at Imperial, and neonatal mice were used for overexpression studies. All mice were housed in specific pathogen-free conditions and given food and water *ad libitum*. Mice were exposed to 20 μg of purified *Alternaria alternata* extract (Greer Laboratories) (in 25μL PBS) intranasally 3 d/wk for 5 weeks. All procedures were conducted in accordance with the Animals (Scientific Procedures) Act 1986.

### Assessment of airway function

AHR in response to increasing doses of methacholine (10-200 mg/mL; Sigma-Aldrich) was measured as previously described,^16^ using the Flexivent system (Scireq, Montreal, Quebec, Canada).

### *In vitro* measurement of airway smooth muscle function

Tracheal tissue was harvested from WT or *Ormdl3* KO^Mer^ mice. Agonist concentration-response curves to methacholine or vehicle were subsequently fitted by least-squares, nonlinear regression based on the Hill equation (Prism 5, GraphPad Software Inc, La Jolla, Calif). Mean EC_50_ values were calculated by averaging data from interpolation of response curves constructed for each individual tissue within a data set.

### Statistical analysis

All data were analyzed using Graph Pad Prism 6 (GraphPad Software). Box and whisker plots depict the median and interquartile range and minimum and maximum values. Line graphs and bar charts are expressed as mean ± SEM, and data were analyzed using nonparametric Mann Whitney *U* tests with significance defined as *P < .05, **P < .01, and ***P < .001.

Methods for *in vitro* assessment of airway smooth muscle function, quantitative PCR, RT-PCR, immunofluorescence, isolation and sorting of epithelial and CD45+ cells, flow cytometry, Western blotting, and mediator analysis are described in the Methods section in this article's Online Repository at www.jacionline.org.

## Results

### *Ormdl3* deficiency protects mice from developing fungal allergen–induced AHR

**Fig 1:**
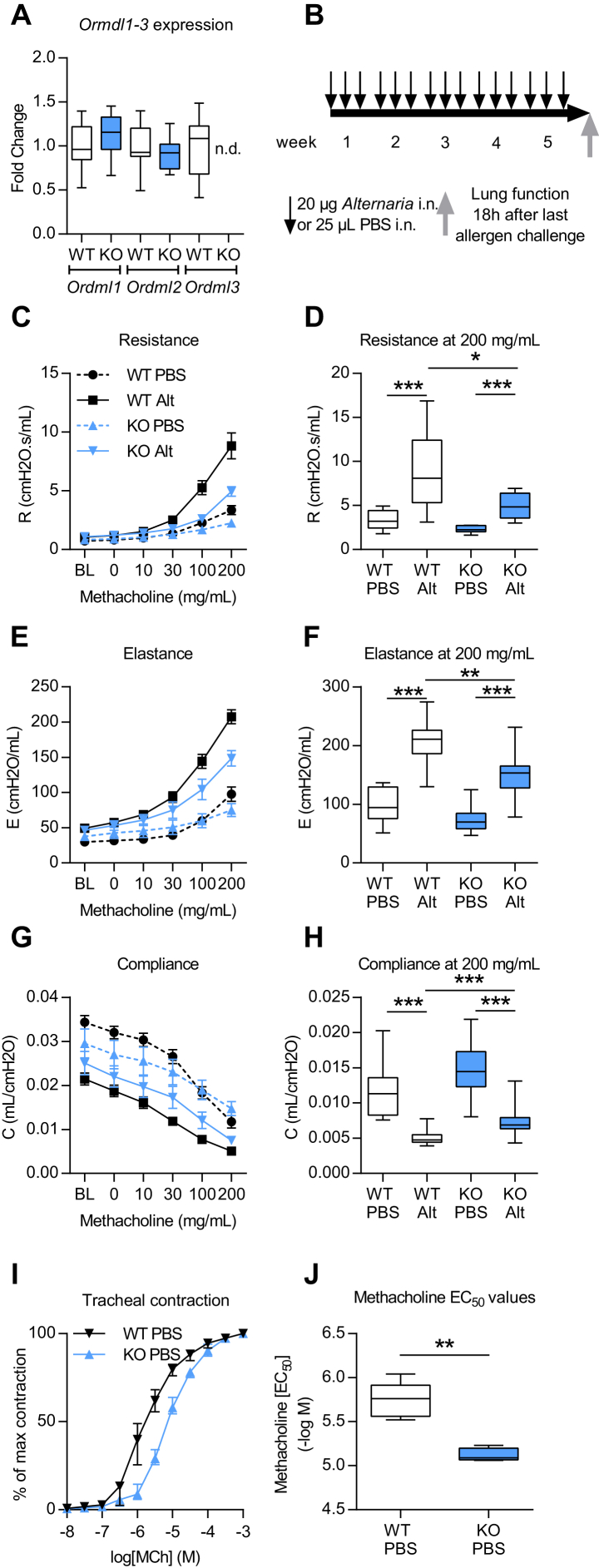
*Ormdl3* KO^Mer^ mice are protected from developing *Alternaria*-induced AHR. **A,** Expression of *Ormdl1* to *Ormdl3* in lungs of naive WT and KO mice by quantitative PCR. **B,** Experimental plan **(C-H)**. Lung function measured using the FlexiVent system. Dose-response curve to methacholine showing (Fig 1, *C*) airway resistance, (Fig 1, *E*) airway elastance, and (Fig 1, *G*) airway compliance. Airway resistance (Fig 1, *D*), airway elastance (Fig 1, *F*), and airway compliance (Fig 1, *H*) at 200 mg/mL methacholine. **I,** Concentration-response curve of tracheas toward increasing concentrations of methacholine. **J,** EC_50_ values of contraction responses. *Alt*, *Alternaria*; *BL*, baseline; *i.n*., intranasal; Data were collected from 2 individually performed experiments. N = 8-12 mice per group. Box and whisker plots depict the median and interquartile range and minimum and maximum values. *P < .05, **P < .005, and ***P < .0005.

ORMDL3 is ubiquitously expressed in adult and fetal tissues including the lung^3^, ^4^ and also in leukocytes.^5^, ^17^ To assess the impact of ORMDL3 on induction of AAD, we generated global *Ormdl3* KO mice. We report that mice lacking *Ormdl3* had no baseline phenotype, appearing healthy and fertile with no alterations in the lung architecture or immune changes at homeostasis compared with WT littermate C57bl/6 mice. Despite deletion of *Ormdl3*, there was no compensatory upregulation of the closely related genes *Ormdl1* and *Ormdl2* in the lungs of the KO mice as determined by quantitative PCR (Fig 1, *A*).

**Fig 2:**
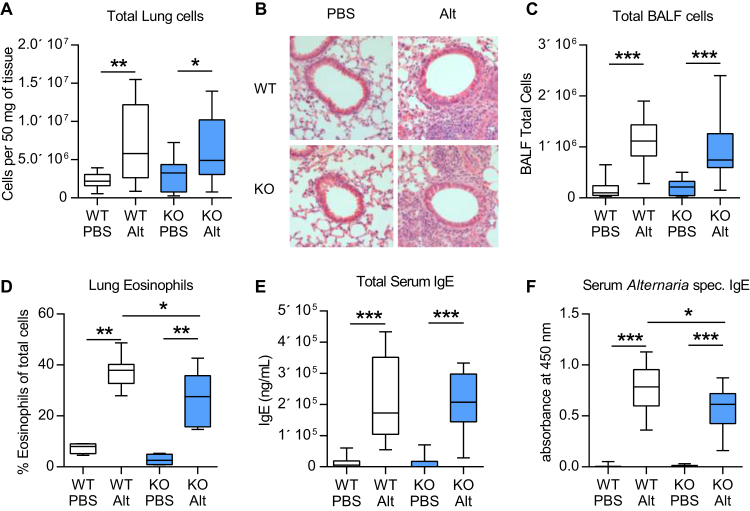
Analysis of *Alternaria*-induced AAD in WT and *Ormdl3* KO^Mer^ mice. **A,** Lung total cell counts. **B,***Alternaria*-induced pulmonary inflammation in tissue sections (H&E staining). **C,** BALF total cell counts. **D,** Eosinophils expressed as the percentage SiglecF^+^ CD11c^−^ CD68^−^ lung cells. **E,** Total IgE concentration. **F,***Alternaria*-specific IgE concentration. Representative photomicrographs are shown. Original magnification ×20. *Alt*, *Alternaria*; *H&E*, hematoxylin & eosin. Data were collected from 2 individually performed experiments. N = 8-16 mice per group. Box and whisker plots depict the median and interquartile range and minimum and maximum values. *P < .05, **P < .005, and ***P < .0005.

**Fig 3:**
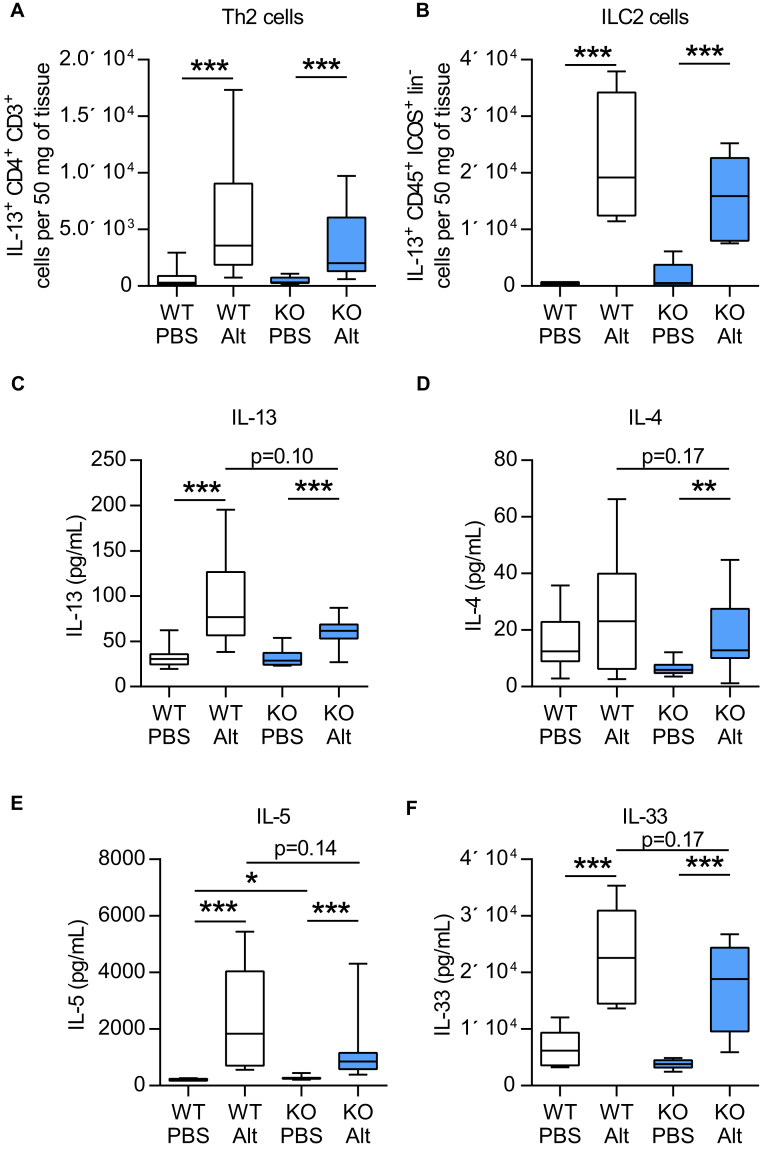
ORMDL3 does not influence *Alternaria*-induced type 2 inflammation or mediators. **A,** Pulmonary T_H_2 cells (IL-13^+^ CD4^+^ CD3^+^). **B,** ILC2 cells (IL-13^+^ ICOS^+^ CD45^+^ lineage^−^ cells). IL-13 **(C)**, IL-4 **(D)**, IL-5 **(E)**, and IL-33 **(F)** cytokine levels in lung tissue determined by ELISA. *Alt*, *Alternaria*. Data were collected from 2 individually performed experiments. N = 8-16 mice per group. Box and whisker plots depict the median and interquartile range and minimum and maximum values. *ILC2*, Type 2 innate lymphoid cell. *P < .05, **P < .005, and ***P < .0005.

Following a single dose of the fungal allergen *Alternaria*, *Ormdl3* mRNA expression has been shown to be induced in bronchial epithelial cells.^5^ To investigate the functional role of ORMDL3 during the development of asthma, we therefore developed a chronic *Alternaria*-induced AAD model (Fig 1, *B*). In WT mice, *Alternaria* induced the hallmark features of asthma, including AHR (Fig 1, *C*-*H*), lung and airway inflammation, as well as eosinophilia (Fig 2, *A*-*D*). IgE concentrations were also augmented following allergen challenge (Fig 2, *E* and *F*). We also observed an increase in pulmonary IL-13^+^ T cells (T_H_2) and type 2 innate lymphoid cells, enumerated by flow cytometry (Fig 3, *A* and *B*). Concomitant with the elevated AHR, pulmonary levels of IL-13 were increased (Fig 3, *C*).

Mice deficient in *Ormdl3* were protected from the development of *Alternaria-*induced airway resistance and elastance (Fig 1, *C*-*F*) and allergen-induced decreases in airway compliance (Fig 1, *G* and *H*). Changes in airway resistance result from altered contractility of the smooth muscle surrounding the airways. Therefore, we chose to investigate whether intrinsic defects in smooth muscle cells could account for differences in lung function between WT and *Ormdl3* KO^Mer^ mice. Tracheal rings from naive mice were isolated and the contraction responses toward increasing methacholine concentrations were compared (Fig 1, *I*). The methacholine EC_50_ value was significantly reduced in mice lacking ORMDL3 (Fig 1, *J*), indicating that ORMDL3 plays a role in airway smooth muscle contractility.

To further dissect the mechanisms that may account for the reduced *Alternaria*-induced AHR in *Ormdl3* KO^Mer^ mice, we profiled the inflammatory cells and mediators present in the lung. *Alternaria* induced cell recruitment to the lung (Fig 2, *A* and *B*) and bronchoalveolar lavage (Fig 2, *C*) was not affected by pulmonary expression of ORMDL3, although the proportion of eosinophils was minimally but statistically significantly reduced (38% vs 28% SiglecF^+^CD11c^−^CD68^−^ cells of total cells) in allergen-exposed *Ormdl3* KO mice^Mer^ (Fig 2, *D*). Class switching to the allergy-associated IgE was unaffected by the loss of ORMDL3 (Fig 2, *E*), although there was a modest decrease in the levels of *Alternaria*-specific IgE (0.78OD vs 0.61OD) (Fig 2, *F*) in *Ormdl3* KO mice^Mer^.

The number of T_H_2 (CD4^+^IL-13^+^) cells and type 2 innate lymphoid cells (Lin^neg^ICOS^+^IL-13^+^) recruited to the lung (Fig 3, *A* and *B*) and bronchoalveolar lavage (data not shown) was not different between *Ormdl3*-sufficient and *Ormdl3*-deficient mice. Despite the reduction in AHR in mice lacking ORMDL3, these mice responded to *Alternaria* exposure with increased levels of pulmonary T_H_2 cytokines including IL-13, IL-4, and IL-5 in the lung (Fig 3, *C*-*E*). Concentrations of these cytokines were less than those observed in WT mice, but the reduction was not statistically significant. A small but significant increase in baseline levels of IL-5 was also noted (204 pg/mL vs 253 pg/mL) in the lung tissue (Fig 3, *E*). Similarly, *Alternaria*-induced increases in the levels of the pulmonary alarmin IL-33 were marginally reduced in *Ormdl3* KO^Mer^ mice (Fig 3, *F*).

Thus, analysis of *Ormdl3* KO^Mer^ mice reveal that lack of ORMDL3 protects against the development of fungal allergen–induced AHR, even though the mice are able to mount a robust T_H_2 response in the lung and periphery.

### *Ormdl3* KO mice have reduced cellular stress responses to *Alternaria*

**Fig 4:**
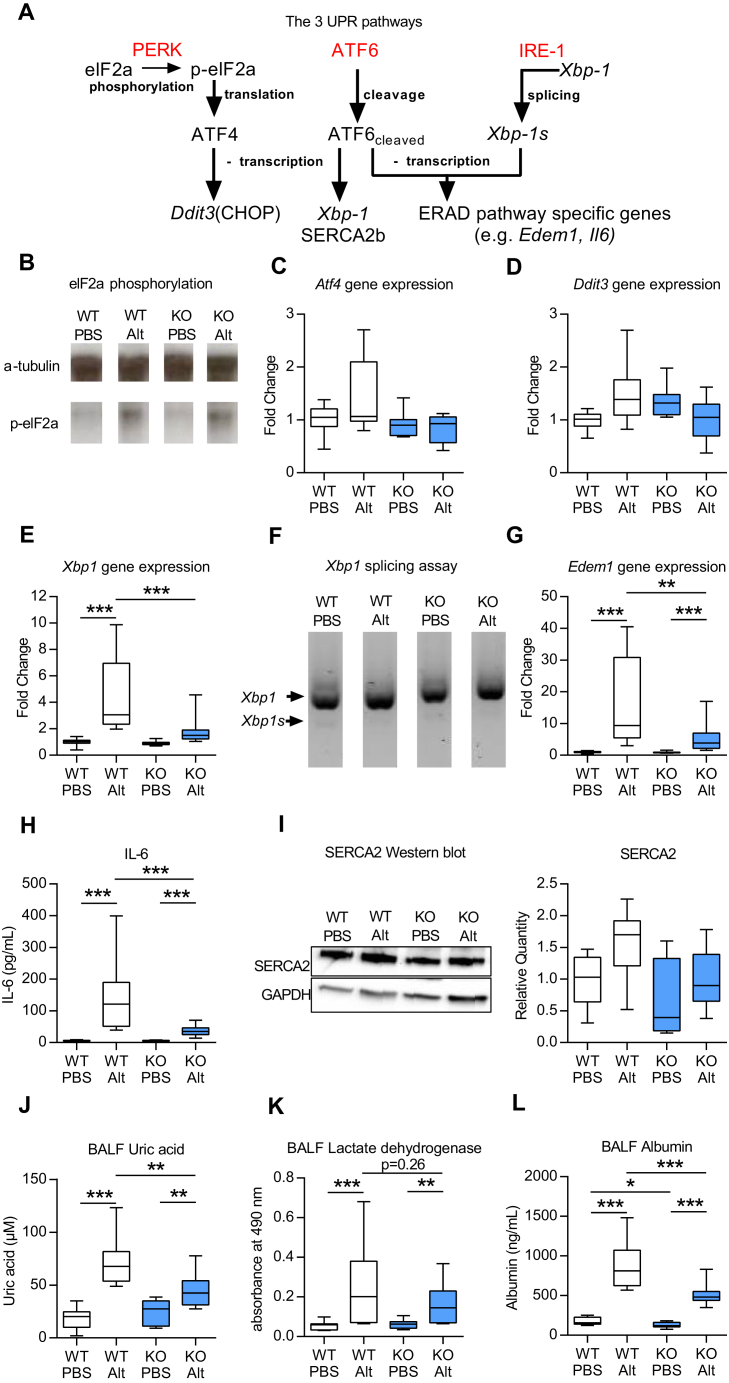
*Ormdl3* KO^Mer^ mice have diminished cellular stress responses to *Alternaria*. **A,** Schematic showing major components of the UPR. **B,** Western blot showing p-eIF2α. Specific quantitative PCR analyses were performed for *Atf4***(C)**, *Ddit3***(D)**, and total *Xbp1***(E)**. **F,** PCR of total and spliced *Xbp1*. **G,***Edem1* quantitative PCR. **H,** IL-6 determined by ELISA. **I,** Western blot showing SERCA2b. Uric acid **(J)**, LDH **(K)**, and albumin **(L)** levels in BALF. *Alt*, *Alternaria*; *Ddit3*, DNA-damage-inducible transcript 3; *Edem1*, ER degradation enhancer, mannosidase alpha-like 1. Data were collected from 2 individually performed experiments. N = 8-16 mice per group. Box and whisker plots depict the median and interquartile range and minimum and maximum values. *P < .05, **P < .005, and ***P < .0005. For analysis of gene expression, Mann-Whitney statistical tests were performed between groups where a greater than 2-fold induction of median values was observed.

*In vitro* and *in vivo* studies have suggested that ORMDL3 facilitates the UPR.^5^, ^9^, ^14^ Therefore, to further investigate how *Ormdl3* deficiency, which does not affect type 2 immune responses in response to chronic *Alternaria* exposure, results in protection from allergen-induced AHR, the UPR was assessed. We specifically examined whether *Alternaria* promotes ER stress, and consequently UPR signaling in the lung, and more importantly, whether the magnitude of this response is altered in *Ormdl3* KO mice. ER stress leads to detachment of the ER chaperone binding immunoglobulin protein from the 3 key ER stress proteins PERK, ATF6, and inositol requiring ER-to-nucleus signal kinase (IRE)-1α, thus initiating 3 parallel pathways of the UPR signaling cascade (Fig 4, *A*).^11^, ^12^ IRE-1α and PERK homodimerise and become activated following autophosphorylation of their respective cytoplasmic domains.^11^ ATF6 is cleaved by proteases in the Golgi complex and subsequently translocates to the nucleus and acts as a transcription factor.^18^ Activated PERK phosphorylates eIF2α, resulting in translational arrest of most proteins. Treatment of mice with *Alternaria* resulted in phosphorylation of eIF2α; however, generation of p-eIF2α was not modulated by ORMDL3 (Fig 4, *B*). Phosphorylation of eIF2α also selectively induces the transcription factor ATF4,^19^ which subsequently increases the expression of DNA-damage-inducible transcript 3, the gene encoding CCAAT/enhancer-binding protein-homologous protein.^20^ Baseline expression of *Atf4* mRNA and DNA-damage-inducible transcript 3 was similar between WT and *Ormdl3* KO^Mer^ mice and was not modulated by exposure of mice to *Alternaria* (Fig 4, *C* and *D*).

The ATF6- and IRE-1–mediated signaling cascades result in activation of the endoplasmic reticulum–associated protein degradation pathway. Acting as a transcription factor, cleaved ATF6 increases the expression of a second transcription factor X-box binding protein 1 (*Xbp1*).^21^ Interestingly, we observed an upregulation of total *Xbp1* mRNA expression in WT mice in response to *Alternaria* (Fig 4, *E*). This was not observed in *Ormdl3* KO^Mer^ mice. IRE-1α splices *Xbp1* mRNA^21^; however, there was very little generation of the spliced *Xbp1* gene product in any of the groups (Fig 4, *F*), indicating that *Alternaria* inhalation does not activate this pathway. Activation of ATF6 induces the transcription of genes that contribute to the clearance of unfolded/misfolded protein from the ER by binding to ER stress response elements. ER degradation enhancer, mannosidase alpha-like 1 (a protein degradation factor)^22^ is an example of an ATF6-inducible gene and its expression was increased in *Alternaria-*treated WT mice compared with PBS controls whereas transcription of this gene in response to allergen challenge in *Ormdl3* KO^Mer^ mice was significantly reduced (Fig 4, *G*). The UPR is also linked to the production of several inflammatory cytokines and ATF6 binds to cAMP response elements promoting IL-6 mRNA transcription.^23^ Exposure of WT mice to *Alternaria* induced a robust IL-6 response in WT mice and this was significantly blunted in *Ormdl3* KO^Mer^ mice (Fig 4, *H*). *In vitro* and *in vivo* studies have demonstrated that ORMDL3 overexpression results in increased expression of another ATF6 target gene, *Atap2a2*, that encodes SERCA2b.^5^, ^9^ However, using *Ormdl3*-deficient mice we show that ORMDL3 is not an absolute requirement for the expression of SERCA2b because basal and *Alternaria*-induced SERCA2b levels were not significantly different between WT and *Ormdl3* KO^Mer^ mice (Fig 4, *I*). Thus, although the UPR is not affected by the absence of ORMDL3 under homeostatic conditions, ATF6 signaling as indicated by increased expression of ER degradation enhancer, mannosidase alpha-like 1 and IL-6 is significantly impaired in pathological conditions where ER stress is induced by exposure to *Alternaria*. Thus, the data indicate that ORMDL3 is involved in the regulation of key genes and proteins induced by ATF6 during ER stress.

There is also a UPR-independent branch of the ER stress response that regulates activation of the NLRP3 inflammasome.^24^ *Alternaria* exposure resulted in increased release of the damage-associated molecular pattern, uric acid. Concomitant with the reduced UPR in *Ormdl3* KO^Mer^ mice, ORMDL3 deficiency also protected against UPR-independent cellular stress as evidenced by the significant reduction in uric acid released in these mice compared with WT animals (Fig 4, *J*). ORMDL3 specifically affects the ER stress pathway since levels of lactate dehydrogenase release from cells, a marker of nonspecific cellular damage, although a modest increase in response to *Alternaria* was not modulated by lack of ORMDL3 (Fig 4, *K*). Concurrent with the decrease in allergen-induced uric acid levels, the *Ormdl3* KO^Mer^ mice also had significantly lower levels of bronchoalveolar lavage fluid (BALF) albumin following *Alternaria* challenge. This is indicative of maintained epithelial integrity and barrier function in *Ormdl3* KO^Mer^ mice compared with WT animals (Fig 4, *L*). Further studies are needed to determine whether induction of cellular stress is an association or causally related to induction of AHR.

### Overexpression of ORMDL3 promotes allergen-induced AHR

**Fig 5:**
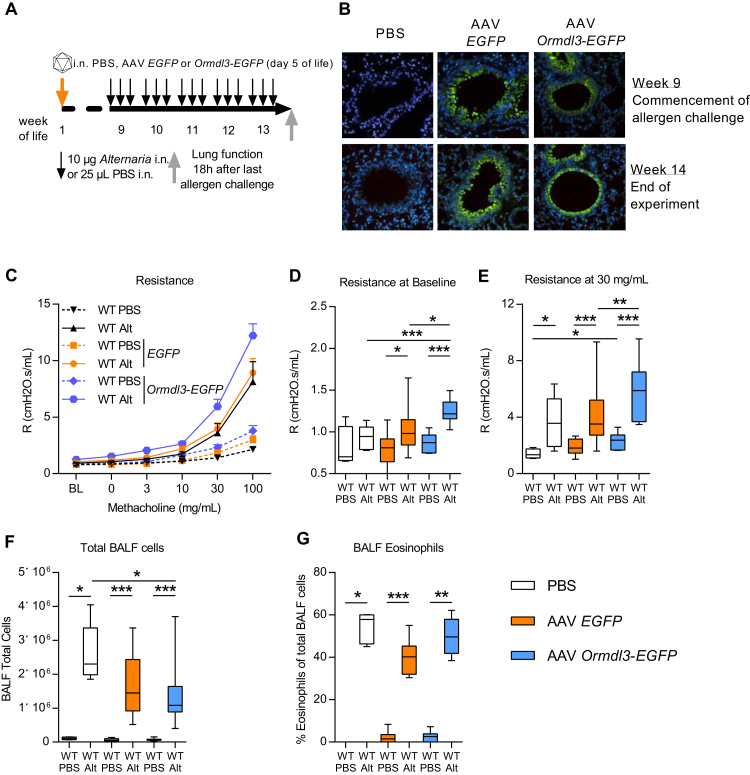
ORMDL3 overexpression in airway epithelial cells enhances AHR. **A,** Experimental plan. **B,** GFP expression (staining green) in the lungs. **C-E**, Lung function measured using the FlexiVent system. Fig 5, *C*, Dose-response curve to methacholine showing airway resistance. Airway resistance at baseline (Fig 5, *D*) and 30 mg/mL methacholine (Fig 5, *E*). **F,** Total cells in the BALF. **G,** Eosinophils. *Alt*, *Alternaria*; Representative photomicrographs are shown. Original magnification ×20. Data were collected from 3 individually performed experiments. N = 8-12 mice per group. Box and whisker plots depict the median and interquartile range and minimum and maximum values. *P < .05, **P < .005, and ***P < .0005.

We next investigated the effect of overexpression of ORMDL3 in the lung using an AAV. Comparative experiments using different AAV serotypes (1, 2, 5, 6, 7, 8, 9, and rh10) encoding the marker gene *EGFP* were conducted. These revealed that intranasal treatment of neonatal mice in the first week of life with AAV9, in combination with neuraminidase to reveal galactose residues on the apical surface of conducting airway epithelial cells to increase AAV9 binding,^25^ resulted in optimal levels of EGFP expression in the lung (data not shown). Because of the lack of available ORMDL3-specific antibodies to track expression, an *Ormdl3-EGFP* gene fusion was introduced into the plasmid pZac2.1 to generate an AAV9 *Ormdl3-EGFP* virus (AAV *Ormdl3*). Five-day-old Balb/c mice were treated with vehicle (PBS), AAV *EGFP*, or AAV *Ormdl3*. At age 8 weeks, (adult) mice were exposed to *Alternaria* for 5 weeks and parameters of AAD were assessed (Fig 5, *A*). A lower dose of *Alternaria* was specifically chosen to determine any potential exacerbation of disease parameters in the mice overexpressing epithelial ORMDL3.

Treatment of neonatal mice with AAV *EGFP* and AAV *Ormdl3* resulted in robust levels of GFP expression primarily in bronchiolar epithelial cells and this expression persisted into adulthood (Fig 5, *B*). There was no effect of treating neonatal mice with the control AAV *EGFP* vector and these animals were phenotypically indistinguishable from PBS-treated littermate controls subsequently exposed to either PBS or *Alternaria* (Fig 5, *C*-*G*). At homeostasis, overexpression of ORMDL3 did not affect either parameters of lung function or the inflammatory profile of the mice (Fig 5, *C*-*G*). However, overexpression of ORMDL3 had a significant effect on *Alternaria*-induced AHR (Fig 5, *C*). At baseline (before the MCh challenge), *Alternaria*-exposed AAV *Ormdl3* mice had significantly higher airway resistance than did allergen-exposed PBS or AAV *EGFP*-treated mice (Fig 5, *D*). Airway responsiveness to MCh challenge was also increased in the mice overexpressing ORMDL3 (Fig 5, *E*). In contrast, recruitment of cells to the airways was not affected by ORMDL3 overexpression (Fig 5, *F* and *G*). *Alternaria*-induced uric acid release was elevated in the ORMDL3-overexpressing mice although the increase was not statistically significant (data not shown).

### Epithelial-specific ORMDL3 expression restores *Alternaria*-induced AAD

**Fig 6:**
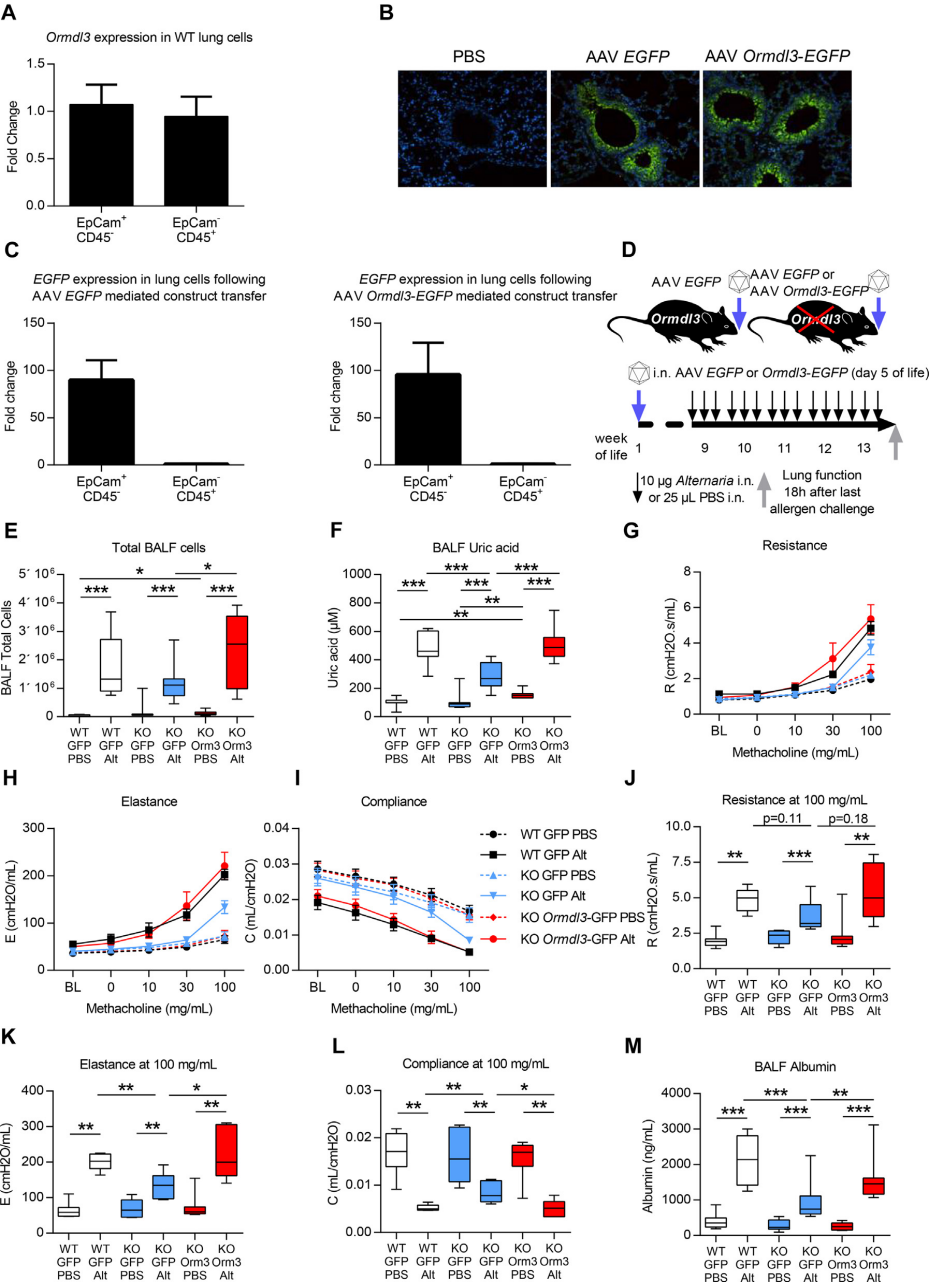
Epithelial ORMDL3 expression drives susceptibility to *Alternaria*-induced AHR. **A,***Ormdl3* expression in epithelial (EpCam^+^) cells and leukocytes (CD45^+^) sorted from WT mice. **B,** GFP expression (staining green) in the lungs. **C,***EGFP* expression in sorted epithelial cells and leukocytes from *Ormdl3* KO^Har^ mice following administration of either AAV *EGFP* or AAV *Ormdl3*. **D,** Experimental plan. **E,** Total cells in the BALF. **F,** Uric acid concentration. **G-L,** Lung function measured using the FlexiVent system. Airway resistance (Fig 6, *G* and *J*), airway elastance (Fig 6, *H* and *K*), and airway compliance (Fig 6, *I* and *L*). **M,** Albumin levels determined in BALF. *Alt*, *Alternaria*; *i.n*., intranasal. Representative photomicrographs are shown. Original magnification ×20. Data were collected from 2 individually performed experiments. N = 7-12 mice per group. Box and whisker plots depict the median and interquartile range and minimum and maximum values. *P < .05, **P < .005, and ***P < .0005.

The previous series of experiments indicated that overexpression of ORMDL3 regulates lung function. Many of the recently identified asthma susceptibility genes including *ORMDL3* are expressed in epithelial cells.^3^, ^5^ In WT mice, *Ormdl3* is equally expressed by epithelial (EpCam^+^) and hemopoietic (CD45^+^) cells (Fig 6, *A*). To assess the particular contribution of epithelial-derived ORMDL3 expression to the generation of *Alternaria-*induced pathology, we reconstituted ORMDL3 expression in airway epithelial cells of *Ormdl3* KO^Har^ mice. Intranasal instillation of AAV *Ormdl3* to neonatal mice resulted in expression almost exclusively in bronchial epithelial cells of the conducting airways as shown by immunofluorescence (Fig 6, *B*) and confirmed by quantitative PCR of sorted epithelial (EpCam^+^) and hemopoietic cells (CD45^+^) (Fig 6, *C*).

**Fig E2:**
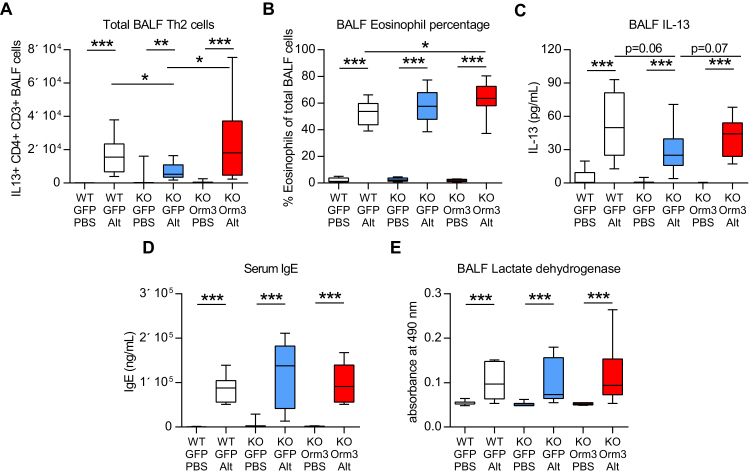
Effect of epithelial ORMDL3 expression on type 2 inflammation. **A,** T_H_2 cells (IL-13^+^ CD4^+^ CD3^+^) in the BALF. **B,** Eosinophils expressed as the percentage SiglecF^+^ CD11c^−^ CD68^−^ in the BALF. **C,** IL-13 levels in the BALF. Serum IgE levels **(D)** and lactate dehydrogenase levels **(E)** in the BALF. *Alt*, *Alternaria*. Data were combined from 2 individually performed experiments. N = 7-12 mice per group. Box and whisker plots depict the median and interquartile range and minimum and maximum values. *P < .05, **P < .005, and ***P < .0005.

At age 8 weeks, WT, KO^Har^, and KO^Har^ mice expressing ORMDL3 specifically in the airway epithelium were exposed to *Alternaria* for 5 weeks and parameters of AAD were assessed (Fig 6, *D*). Expression of epithelial ORMDL3 was associated with a small but statistically significant increase in total cells in the BALF (WT GFP PBS 6.5 × 10^4^ vs KO *Ormdl3* PBS 9.8 × 10^4^) and uric acid (WT *EGFP* PBS 99 μM and KO *EGFP* PBS 90 μM vs KO *Ormdl3* PBS 149 μM) in the airways but there was no other baseline phenotype in the absence of allergen challenge (Fig 6, *D* and *E*, and Fig E2). Restoration of epithelial ORMDL3 expression in the KO^Har^ mice resulted in enhanced *Alternaria-* induced AHR that was equal in magnitude to that recorded in WT mice, implying that epithelial-derived ORMDL3 governs the deleterious *Alternaria*-induced changes in lung function (Fig 6, *G*-*L*). The total number of leukocytes recruited to the airway lumen following *Alternaria* challenge was increased in the KO mice expressing epithelial ORMDL3 compared with *Ormdl3*-deficient mice (Fig 6, *E*). Low-dose *Alternaria* revealed an effect on T_H_2 cells, with reduced recruitment in *Ormdl3* KO^Har^ mice (Fig E2, *A*). Epithelial expression of ORMDL3 in KO^Har^ mice reestablished the allergen-induced increase in T_H_2 cell accumulation (Fig E2, *A*). There was also a small but significant increase in airway eosinophilia in the epithelial ORMDL3-expressing KO^Har^ mice compared with WT mice (Fig E2, *B*). Congruent with the observed changes in type 2 inflammation, BALF IL-13 levels were reduced in *Ormdl3* KO^Har^ mice although the reduction was not statistically significant (Fig E2, *C*). IL-13 concentrations in *Ormdl3* KO^Har^ mice reconstituted with epithelial ORMDL3 were not different from those in WT mice. There was no effect of ORMDL3 on total IgE (Fig E2, *D*) or *Alternaria*-specific IgE levels in allergen-exposed mice (data not shown). Levels of the cell damage marker lactate dehydrogenase were similar in all groups of mice exposed to allergen (Fig E2, *E*), congruent with our previous observations. Viral expression of ORMDL3 in KO^Har^ mice restored the uric acid response to *Alternaria* (Fig 6, *F*). In response to allergen, levels of BALF albumin were decreased in *Ormdl3* KO^Har^ mice compared with WT mice, indicative of increased epithelial integrity (Fig 6, *M*). However, in the KO^Har^ mice reconstituted with epithelial ORMDL3, albumin levels were equivalent to those in WT mice (Fig 6, *M*). These data show that epithelial ORMDL3 governs the cellular stress response and AHR induced by inhaled *Alternaria*.

## Discussion

GWAS have established a strong correlation between SNPs at the 17q21 locus and the development of asthma and *ORMDL3* has been proposed as an asthma susceptibility gene. However, direct *in vivo* evidence for a pathophysiological function of ORMDL3 in a long-term model of AAD induced by a relevant aeroallergen associated with human disease is lacking.

ORMDL3 expression did not influence the number of T_H_2 cells or type 2 innate lymphoid cells recruited to the lung in response to *Alternaria*; however, eosinophilia was reduced in mice lacking ORMDL3. These data are in agreement with published literature demonstrating that ORMDL3 regulates eosinophil trafficking, recruitment, and degranulation.^17^ However, in our lower dose *Alternaria* exposure regime used in the epithelial ORMDL3 reconstitution experiments (Fig 6), we show that the total number of inflammatory cells recruited to the airway lumen are increased in mice expressing epithelial ORMDL3 compared with the global KO animals. The increased BAL cellularity was due to increased numbers of T_H_2 cells and eosinophils, indicating that epithelial ORMDL3 facilitates the trafficking of these cells to the airway lumen. However, the ORMDL3-dependent element of allergen-induced inflammation is overcome with higher doses of *Alternaria*. Levels of total IgE were unaffected by the loss of ORMDL3, indicating that ORMDL3 primarily affects innate rather than adaptive immunity in the generation of AAD.

Our data show that *Ormdl3* KO mice are protected from the development of increased airway resistance, a function of smooth muscle contractility, following prolonged *Alternaria* exposure, implying that ORMDL3 is a critical driver of this aspect of AHR. Interestingly, although ORMDL3 is pivotal for the generation of *Alternaria-* induced AHR, the same phenotype was not observed in response to the aeroallergen house dust mite (data not shown). *Ormdl3* KO mice develop AAD when exposed to house dust mite and the magnitude of AHR is equivalent to allergen-treated WT mice. This suggests that ORMDL3 mediates an *Alternaria*-specific activation of AAD. Sensitization to *Alternaria* has been implicated in severe asthma risk and fungal exposure is known to be associated with an increase in life-threatening exacerbations of the disease.^26^, ^27^ Intriguingly, SNPs in the chromosome 17q21 region in addition to being reproducibly associated with asthma have been linked to asthma exacerbations requiring hospitalization and/or oral steroids.^28^, ^29^

Mice globally overexpressing ORMDL3 have previously been shown to have elevated levels of SERCA2b, which transports calcium from the cytosol to the ER.^5^, ^9^ However, expression of SERCA2b is not solely dependent on the presence of ORMDL3 because levels were not altered in *Ormdl3*-deficient mice. ORMDL3 has also been shown to inhibit the activity of SERCA2b and this modulatory effect is suggested to result from direct association of the 2 proteins.^14^ The reduced sensitivity of the smooth muscle from *Ormdl3* KO mice to pharmacologically induced contraction may therefore be due to decreased intracellular calcium concentrations as a result of increased SERCA2b-mediated uptake to the ER in the absence of ORMDL3, and reduced activity of SERCA in airway smooth muscle is thought to contribute to airway remodeling in those with moderate/severe asthma.^30^ ORMDL3, which has been shown to be induced by *Alternaria*, also regulates ceramide biosynthesis, and high-level expression of ORMDL3 increases ceramide production.^8^ Allergen induces elevated ceramide levels with concomitant AHR, which is ablated in mice treated with the sphingosine analogue FTY 720, which reduces both ORMDL3 and ceramide levels.^8^ Thus, the reduced smooth muscle contractility measured in *Ormdl3* KO mice may also result from dysregulated lipid homeostasis.

We show that ORMDL3 expression specifically in bronchiolar epithelial cells restores the *Alternaria*-induced increase in AHR, suggesting that although ORMDL3 may have a direct effect on airway smooth muscle, it can also act indirectly. Uric acid levels are increased in patients with asthma following segmental allergen challenge and treatment of HDM-challenged mice with uricase ablates allergen-induced AHR.^31^ We have shown that *Alternaria*-induced secretion of uric acid into the airways is blunted in *Ormdl3* KO mice. Conversely, viral-mediated expression of ORMDL3 in airway epithelial cells of KO mice reinstated the uric acid response to allergen challenge, clearly demonstrating the importance of epithelial-derived ORMDL3 in mediating *Alternaria*-induced release of damage-associated molecular patterns. Interestingly, uric acid is released early in the immune response to protease allergens,^32^ and levels can be used as a biomarker for the severity of asthma exacerbations in patients.^33^ Epithelial cells have an active uric acid transport system, and basal secretion of uric acid by human airway epithelial cells has been demonstrated.^34^ Uric acid is known to have direct effects on smooth muscle cells including stimulating endothelin-1 expression and increased intracellular calcium concentrations, which can induce smooth muscle contraction.^35^, ^36^ Thus, epithelial-expressed ORMDL3 can modulate lung function via its effects on uric acid release induced by allergen inhalation.

Many of the recently identified asthma susceptibility genes, including *ORMDL3*, are known to be expressed in epithelial cells,^3^, ^5^ and disrupted airway epithelial barrier function is thought to be a critical controller of disease induction.^2^ Using *Ormdl3* KO mice, we can infer that this protein has a vital role in the epithelial response to allergen and dysregulated expression of ORMDL3 likely contributes to a reduction in epithelial integrity as demonstrated by the increase in bronchoalveolar lavage albumin levels in ORMDL3-sufficient mice compared with KO animals. In KO mice expressing epithelial ORMDL3, *Alternaria*-induced increases in luminal albumin were restored, suggesting that epithelial ORMDL3 governs allergen-induced loss of epithelial integrity.

Another branch of the ER stress response is the UPR. The protein sensors PERK, IRE-1α, and ATF6 respond to ER stress by increasing the expression of proteins such as binding immunoglobulin protein and CCAAT/enhancer-binding protein-homologous protein,^37^ which have been shown to be elevated in patients with asthma and in a murine model of neutrophilic, steroid-resistant AAD induced by LPS/ovalbumin. These markers are positively correlated with AHR and therapeutic treatment of mice with 4-phenylbutyric acid, a potent ER stress inhibitor, has been shown to reduce AHR, implying that activation of the UPR, which is dependent on ORMDL3, is intimately linked with lung function. In the lung, ORMDL3 activates the UPR via the ATF6 pathway, increasing transcription of endoplasmic reticulum–associated protein degradation pathway-specific genes (Edem-1) and regulating the expression of IL-6, which has been implicated in the pathogenesis of asthma.^13^

It is not known how the levels of ORMDL3 expression achieved by AAV-mediated gene transfer to the airway epithelium compare with the *in vivo* levels in WT mice due to the lack of specific reagents to differentiate between ORMDL1, 2, and 3 isoforms because of the high level of homology between these proteins, which we have shown directly using KO mice. Despite this, the data clearly demonstrate that epithelial ORMDL3 is essential for the generation of AAD and GWAS indicate that only the ORMDL3 isoform is associated with asthma susceptibility. ORMDL3 has a cAMP response element (CRE) in the promoter region and protein kinase A–dependent cAMP response element binding protein phosphorylation results in binding to the cAMP response element and initiation of *ORMDL3* transcription.^38^ ORMDL3 activates ATF6, increasing the transcription of cAMP response element responsive genes; thus, ORMDL3 may mediate its own expression via a positive feedback loop. In addition, Alternariol, a mycotoxin of *Alternaria*, increases cAMP response element binding protein binding to CREs^39^ and this may be the mechanism by which *Alternaria* increases ORMDL3 expression.

Specific clinical asthma phenotypes (recurrent severe exacerbations) have previously been shown to be associated with distinct genotypes (cadherin regulated family member 3, locus).^40^ Stratifying patients with severe asthma into 2 groups on the basis of their response to fungal exposure reveals that the patients who respond to fungal allergens have earlier onset symptoms and require more oral steroid therapy than do those who do not and experience a greater number of asthma exacerbations.^41^, ^42^, ^43^ GWAS data predict that elevated ORMDL3 expression increases the risk of severe asthma and asthma exacerbations; thus, given the association between ORMDL3 and *Alternaria*-induced AHR revealed in the present study, it would be pertinent to determine whether SNPs in the 17q21 locus are associated with severe fungal-associated asthma phenotypes. With the current emphasis on personalized medicine, confirmation of the mechanisms and therapeutic targets in these specific patients could offer tailored treatments for this group of patients with asthma who frequently present with severe disease and are resistant to current steroid treatments. The ER stress inhibitor sodium phenylbutyrate (4-phenylbutyric acid) is licensed for oral administration and used therapeutically in the management of inherited urea cycle disorders.^44^ Because of its additional chaperone function it would be of interest to determine whether this drug also has efficacy in patients with asthma carrying genetic variations in the *ORMDL3* locus.

In conclusion, ORMDL3 is a crucial regulator of smooth muscle contractility and subsequent AHR, acting potentially via cellular stress pathways, the UPR, and uric acid release. Modulation of cellular stress may represent a novel therapeutic avenue for the treatment of asthma.

**Key messages**

• In response to fungal exposure, *Ormdl3* mediates the ATF6-dependent pathway of the UPR and uric acid release.
• The cellular stress promoted by *Ormdl3* orchestrates AHR during allergic immune responses.
• Epithelial ORMDL3 is crucial for the induction of airway resistance.

## Methods

### RT-PCR

Lung tissue samples were homogenized in TRIzol solution (Life Technologies, Warrington, UK) followed by addition of chloroform to extract nucleic acids. RNA was isolated using the RNeasy Mini Kit (Qiagen, Manchester, UK), including an on-column DNase digest with an RNase-free DNase set (Qiagen) as per manufacturer's instructions. cDNA was amplified with random primers and the High-Capacity cDNA Reverse Transcription Kit (Life Technologies) according to the manufacturer's instructions.

Real-time RT-PCR reactions were performed on a ViiA 7 Real-time PCR System (Life Technologies) in conjunction with TaqMan Fast Advanced Master Mix (Life Technologies) and following TaqMan Gene Expression Assays (Life Technologies): *Atf4* (Mm00515325_g1), DNA-damage-inducible transcript 3 (Mm01135937_g1), ER degradation enhancer, mannosidase alpha-like 1 (Mm00551797_m1), *Xbp1* (Mm00457357_m1), *Gapdh* (Mm99999915_g1), and *Hprt* (Mm01545399_m1). Expression of each target gene was normalized to the arithmetic mean of the 2 housekeeping genes *Gapdh* and *Hprt*. Data were then expressed as fold-change of relative mRNA expression in experimental groups versus naive WT animals.

*Xbp-1* and spliced *Xbp-1* (*Xbp-1s*) transcripts were amplified by PCR (forward primer: ACACGCTTGGGAATGGACAC; reverse primer: CCATGGGAAGATGTTCTGGG). The products were separated using a 4% agarose gel (Ultrapure agarose; Invitrogen, Waltham, Mass).

### Isolation and sorting of CD45^+^ EpCam^−^ and CD45^−^ EpCam^+^ cells

Airway epithelial cells were sorted as previously reported,^E1^ with minor amendments. Lungs were perfused with ice-cold PBS through the right cardiac ventricle. Dissociation solution (1.5 mL) (Dulbecco modified Eagle medium/F12 [no phenol red; Life Technologies]) substituted with 25 mM HEPES (Sigma-Aldrich, Dorset, UK), penicillin/streptomycin, and 5 mg/mL dispase II (Sigma-Aldrich) was injected into the lungs through the trachea using a 1-mL syringe, followed by 0.5 mL of 1% low gelling temperature agarose (Sigma-Aldrich). The trachea was consequently closed using a hemostat and the chest was cooled down with ice to facilitate agarose solidification. The lung lobes were then harvested and incubated at 37°C for 1 hour in 5-mL dissociation solution under slight agitation. The digested tissue was passed through a 100-μm cell strainer (BD, Oxford, UK), cells were precipitated by centrifugation, washed through a 100-μm cell strainer for a second time to remove any remaining, solidified agarose, and incubated at room temperature for 10 minutes in Dulbecco modified Eagle medium/F12/25 mM HEPES in the presence of 50 μg/mL DNase I (Roche, Welwyn Garden City, UK). Erythrocytes were lysed and the cell suspension was extracellularly stained in sorting buffer (1% BSA in PBS) with phycoerythrin-conjugated EpCam (eBioscience, Cheshire, UK) and Brilliant Violet 421–conjugated CD45 (Biolegend) antimouse antibodies and sorted using a BD FACSAria III cell sorter. QIAshredder and RNeasy micro kits (Qiagen) were used in combination to isolate RNA. cDNA was prepared using the SuperScript VILO Master Mix (ThermoFisher, Waltham, Mass). RNA/cDNA preparation steps were performed according to manufacturers' instructions. Transcript levels of *EGFP* were assessed by quantitative RT-PCR using *EGFP*-targeting Taqman gene expression assays (Mr04329676_mr; Thermo Fisher). *Gapdh* served as the reference gene.

### Immunofluorescence

Paraffin sections were stained sequentially with rabbit anti-GFP antibody (Abcam, Cambridge, UK), biotinylated goat anti-rabbit (Jackson, Suffolk, UK), and streptavidin AlexaFluor488 (Molecular probes, Invitrogen). Tissue sections were visualized and images overlayed using a LeicaDM2500 microscope and QWin software (Leica Microsystems [UK] Ltd, Milton Keynes, UK).

### Western blot

Lung tissue was homogenized at 50 mg/mL tissue in RIPA buffer (Sigma, Dorset, UK) followed by centrifugation at 13,000 rpm for 10 minutes at 4°C. The resultant supernatant was used for Western blot. Proteins were separated by SDS-PAGE and transferred to polyvinylidene difluoride membranes. Membranes were blocked with 5% (w/v) nonfat milk in TRIS-buffered saline with 0.05% tween-20. The following primary antibodies were used: rabbit monoclonal anti-SERCA2 (Abcam), rabbit polyclonal anti-GAPDH (Millipore, Watford, UK), rabbit polyclonal anti-GAPDH Phospho-eIF2α (Ser51) (Cell Signaling Technology, Leiden, The Netherlands), and rabbit monoclonal anti-alpha tubulin antibody (Abcam).

### Flow cytometry

BALF recovery, lung tissue digest, cell counting, and differential cell counting were performed as described previously.^E2^

Single-cell suspensions from lung tissue and bronchoalveolar lavage were stimulated with 40 ng/mL PMA (Sigma-Aldrich) and 3 μg/mL ionomycin (MERCK Millipore, Watford, UK) in RPMI/10% FCS containing 10 μg/mL Brefeldin A (Sigma-Aldrich) at 37°C, 0.5% CO_2_ for 3 hours. Cells were then washed and incubated for 20 minutes at room temperature with 10 μL normal rabbit serum (Sigma-Aldrich). After washing, cells were stained for extracellular antigens in 5% FCS/1% BSA in PBS for 30 minutes at 4°C. Cells were then washed, fixed, and permeabilized using Fix/Perm kit (eBioscience, Hatfield, UK) before being stained for intracellular antigens. All antibodies were purchased from eBioscience except anti-ICOS (Biolegend, London, UK). Acquisition was performed using Fortessa (BD) and analysis with FlowJo software (Flowjo, Ashland, Ala).

### Mediator analysis

Lung tissue was homogenized in HBSS (Life Technologies) containing protease inhibitor cocktail (Roche). Lung tissue supernatant, bronchoalveolar lavage, and serum were analyzed by ELISA; IL-4 and IL-5 (BD PharMingen, Oxford, UK), IL-13 (Mouse Quantikine Kit [R&D Systems, Abingdon, UK]), IL-33 (Mouse IL-33 DuoSet ELISA, R&D Systems), and albumin (Bethyl Laboratories Inc, Montgomery, Tex). Paired antibodies for IgE (R&D Systems) were used to measure serum immunoglobulin levels. For determination of *Alternaria-*specific IgE levels, high-binding ELISA plates were coated with 50 μg/mL *Alternaria* extract. Uric acid levels were determined by Amplex Red uric acid assay (Life Technologies) and lactate dehydrogenase levels by a lactate dehydrogenase–based, in vitro toxicology assay kit (Sigma-Aldrich), according to the manufacturers' instructions.

### *In vitro* measurement of airway smooth muscle function

Tracheal tissue was harvested from WT or *Ormdl3* KO^Mer^ mice and bathed in Kreb's Henseleit solution warmed to 37°C and aerated with 95% O_2_/5% CO_2_. The tracheas were then cleared of extraneous tissue and split into 2 rings by cutting along the transverse plane. Tissues were then sutured to force-displacement transducers and placed in tissue baths. Following time to stabilize, tissues were challenged with acetylcholine (1 mM) 3 times to confirm viability and to standardize responses. Cumulative concentration-response curves (10 nM-1 mM) were then generated to methacholine or vehicle (Kreb's Henseleit solution). Agonist concentration-response curves were subsequently fitted by least-squares, nonlinear regression based on the Hill equation (Prism 5, GraphPad Software Inc). Mean EC_50_ values were calculated by averaging data from interpolation of response curves constructed for each individual tissue within a data set.
